# A retrospective narrative review of kidney stone composition in patients from two race groups having different stone occurrence profiles: a hypothesis-generating study of stone formation based on stone composition alone

**DOI:** 10.1007/s00240-026-01987-2

**Published:** 2026-04-21

**Authors:** Allen L. Rodgers, Grace Carmichael

**Affiliations:** 1 Department of Chemistry, University of Cape Town, Cape Town, South Africa; 2 Department of Statistical Sciences, Statistical Consulting Unit, University of Cape Town, Cape Town, South Africa

**Keywords:** Kidney stones, Stone composition, Stone pathogenesis, Inter-race, Inter-gender, Pathophysiological associations

## Abstract

Studies of kidney stone composition have been ongoing for over 60 years but have failed to establish a compelling relationship between stone composition and stone pathophysiology. As such, a focussed investigation involving a different approach to those employed previously is warranted. The stone anomaly in South Africa provides an ideal scenario for such an investigation. In this country, stones occur in the white population group (W) but historically they have been relatively rare in their black African counterparts (B). Accounting for this anomaly in terms of urine composition has not been convincingly described. We undertook to achieve better understanding of this anomaly and the riddles of stone formation in general by comparing compositions and their proportionate presence in stones formed in these groups. Composition of 3783 stones were retrieved from stone composition files in our research laboratory. Each was analysed by x-ray powder diffraction and was classified according to the race and gender of the patients from whom they were obtained and according to the presence of a main component, defined as constituting ≥ 50% of the overall composition. Chi-squared tests were used to test differences in proportions between the groups and for correlation p-values. Results were adjusted for multiple-comparisons using a Bonferroni correction of the p-values. Phi coefficients and p-values were calculated using functions from the R-library. Correlation tables were generated using the R library *‘flextable’*, while correlation plots were generated using the *Corrplot* function from the R library *‘DescTools’*. Statistical significance was assigned to correlation coefficients ≥ 0.25, *p* ≤ 0.05. Systematic literature searches were conducted to identify compositional, clinical and pathophysiological associations. The proportion of calcium oxalate dihydrate (COD) stones was significantly lower in B than in W (7.2% vs. 18.8%, *p* = 0.006). Uric acid dihydrate (UAD), sodium urate and ammonium acid urate stones were common in W, but were absent in B. Intergender differences and correlation analyses were unremarkable as they were in alignment with global observations. Literature searches highlighted weaker crystal-cell adhesive forces associated with COD compared to the monohydrate (COM) and relatively slower transformation kinetics of the former to the latter. Associations between UAD, type 2 diabetes, obesity, metabolic syndrome and Randall’s plaque were also featured in these searches. We speculate that smaller proportions of COD in B might be indicative of a mechanism involving the transformation kinetics of COD to COM and the presence of weaker crystal-cell adhesion forces associated with COD. We also hypothesize that the absence of UAD, NaUr and AAU in B is indicative of a loss of promotive capacity for the formation of Randall’s plaque, substantiated by an apparent lower incidence of the latter in B, and a concomitantly lower stone occurrence rate in this group. Finally, we suggest that the South African stone anomaly might be related to the reported lower metabolic syndrome occurrence in B.

## Introduction

Composition of kidney stones as a potential key to understanding their formation has been investigated in major stone collections for many years [1–9] However, these have been mainly observational. Other studies have sought to identify variations in stone type with gender, age, race, ethnicity and regional distributions [10–14]. While these have provided some insights into stone formation per se, they have been of a general nature and have not been exhaustively pursued. Recognising that such studies have been ongoing for over 60 years without establishing a compelling relationship between stone composition and stone pathophysiology, a more focussed investigation involving a different approach is warranted.

In South Africa, a stone anomaly exists between black African (B) and white (W) population groups. Stones have been historically rare in the former but relatively common in the latter. This intrigue is well known, having first been reported nearly 100 years ago [15]. Since then, studies have compared composition in stones from both groups but like stone studies elsewhere, these have been observational rather than interrogative [16–23]. Exploration of compositional variations in stones from these two population groups would be of considerable interest particularly given that the respective stone occurrence rates in the groups are different. Regrettably, there are no recent studies of stone occurrence and composition in South African B and W [24]. However, historical stone data in our laboratory records provide an ideal opportunity for retrospective examination of composition and interrogation of the notion that inter-group differences in such compositions per se might provide an improved understanding of the anomaly and of stone formation more generally.

## Methods

### Stones

Stones were referred to our laboratory by the Urology Departments at Groote Schuur and Tygerberg Hospitals in Cape Town as part of an ongoing analytical service offered by our research group during the period 2000–2008. Each stone was analysed by ourselves using x-ray powder diffraction. Patient demographics were provided by the referring hospitals. Stone compositions were classified according to self-declared gender and race (BM: black African males, BF: black African females, WM: white males, WF: white females). These classifications were assigned in accordance with international and local ethical guidelines for reporting academic research involving human subjects (see the section “Declarations” at the end of this paper). Stones were further classified according to the presence of a main component, defined as constituting ≥ 50% of the overall stone composition.

### Literature

Systematic literature searches were conducted to identify compositional, clinical and pathophysiological associations with stones. These were conducted using Google Scholar and Pubmed. Search words included were kidney stones, associations, chemical composition, calcium oxalate monohydrate, calcium oxalate dihydrate, stone pathophysiology.

### Statistical analysis

To determine whether there were inter-ethnic and inter-gender differences regarding main components, chi-squared tests were used to test the difference in proportions between the groups using correlation p-values. Adjustment for differences in sample size was included. Results were adjusted for multiple-comparisons using a Bonferroni correction of the p-values. Some of the statistical tests were not performed as the sample sizes of the groups were too small. Phi coefficients were calculated using the *phi* function from R library ‘*psych’* and p-values using the *chisq.test* function from the R library ‘stats’. Correlation tables were generated using the R library *‘flextable’*. Correlation plots (heatmaps of the correlation coefficients) were generated using the *Corrplot* function from the R library *‘DescTools’*. A positive correlation was interpreted as indicating that the components were more often both present or both absent in a stone; a negative correlation indicated that the two components showed opposite patterns of presence and absence. Statistical significance was assigned to correlation coefficients ≥ 0.25, p ≤ 0.05.

## Results

A total of 3783 stones was analysed (BM: 80, BF: 31, WM: 2757, WF: 915, comprising 2.1%, 0.8%, 72.9% and 24.2% of the total collection respectively. Stone composition data and *p-* values for inter-gender and inter-race comparisons are given in Table 1.

**Table 1:** Inter-race and inter-gender comparison of the percentage proportion of main stone components in a collection of South African kidney stones

Main Component (≥ 50%)	BLACK AFRICAN SUBJECTS							WHITE SUBJECTS							BLACK-WHITE COMPARISON		
	Males + Females		Males		Females		BM vs. BF	Males + Females		Males		Females		WM vs. WF	BM vs. WM	BF vs. WF	B vs. W
	n	% of B	n	% of BM	n	% of BF	p-value	n	% of W	n	% of WM	n	% of WF	p-value	p-value	p-value	p-value
COM	57	51.4	47	58.8	10	32.3	**0.037**	1951	53.1	1552	56.3	399	43.6	**< 0.001**	~ 1	0.629	~ 1
COD	8	7.2	7	9.5	1	3.3	0.937	692	18.8	558	20.2	134	14.6	**< 0.001**	**0.034**	0.221	**0.006**
BRU	1	0.9	1	1.2	0	0.0		30	0.8	21	0.8	9	1.0	~ 1.000			
APA	7	6.3	2	2.5	5	16.1	**0.024**	292	8.0	135	4.9	157	17.2	**< 0.001**	0.973	~ 1.000	~ 1.000
STR	15	13.5	3	3.8	12	38.7	**< 0.001**	281	7.7	125	4.5	156	17.0	**< 0.001**	~ 1.000	**0.006**	0.070
UA	18	16.2	15	18.6	3	9.6	0.734	535	14.6	452	16.4	83	9.1	**< 0.001**	~ 1.000	~ 1.000	~ 1.000
UAD	0	0.0	0	0.0	0	0.0		50	1.3	45	1.6	5	0.5	**0.042**			
AAU	1	0.9	1	1.2	0	0.0		22	0.6	12	0.4	10	1.1	0.077			
NaUr (SUM)	0	0.0	0	0.0	0	0.0		37	1.0	24	0.9	13	1.4	0.446			
CYS	1	0.9	1	1.2	0	0.0		33	0.9	27	1.0	6	0.7	~ 1.000			
TOTAL	111	100	80	100	31	100		3672	100	2757	100	915	100				

Bold type: statistically significant

Percentages represent the proportion of each component within the respective parent groups listed above

COM: calcium oxalate monohydrate; COD: calcium oxalate dihydrate; Bru: brushite; APA: apatite; STR: struvite; UA: uric acid; UAD: uric acid dihydrate; AAU: ammonium acid urate; NaUr: sodium urate; SUM: sodium urate monohydrate (NaUr and SUM are equivalent); CYS; cystine

We note that there is only one statistically significant difference between B and W, namely the proportion of calcium oxalate dihydrate stones (COD) is lower in B than in W (7.2% vs. 18.8%, *p* = 0.006) and that it is driven by the significantly lower proportion of this component in BM compared to WM (9.5% vs. 20.2%, *p* = 0.034). A similar situation is observed in the proportion of struvite (STR) which tends towards being significantly greater in B than in W (13.5% vs. 7.7%, *p* = 0.070), driven in this case by the significantly larger proportion of this component in BF compared to WF (38.7% vs. 17.0%, *p* = 0.006).

Qualitatively, it is noted that while uric acid dihydrate (UAD) and sodium urate (NaUr) were observed in stones from W, they did not occur in B. Similarly, although ammonium acid urate (AAU) was not uncommon in W, there was only one stone having this composition in B.

Regarding inter-gender comparisons within the same ethnic group, Table 1 shows that there are three statistically significant differences which occur in both race groups. The first is the proportion of calcium oxalate monohydrate (COM) which is significantly greater in M than in F (B: 58.8% vs. 32.3%, *p* = 0.037; W: 56.3% vs. 43.6%, *p* < 0.001). The second is the proportion of apatite (APA) which is significantly lower in M than in F (B: 2.5% vs. 16.1%, *p* = 0.02; W: 4.9% vs. 17.2%, *p* < 0.001). The final common difference is the proportion of STR which is also significantly lower in M than in F (B: 3.8% vs. 38.7%, *p* < 0.001 and 4.5% vs. 17.0%, *p* < 0.001).

Besides these, there are three further inter-gender differences, which occur only in W, thereby simultaneously qualifying as inter- ethnic differences. These refer to the proportions of COD, UA and UAD, all of which are significantly higher in WM than in WF (20.2% vs. 14.6% *p* < 0.001, 16.4% vs. 9.1% *p* < 0.001 and 1.6% vs. 0.5% *p* = 0.042 respectively).

**Fig. 1:**
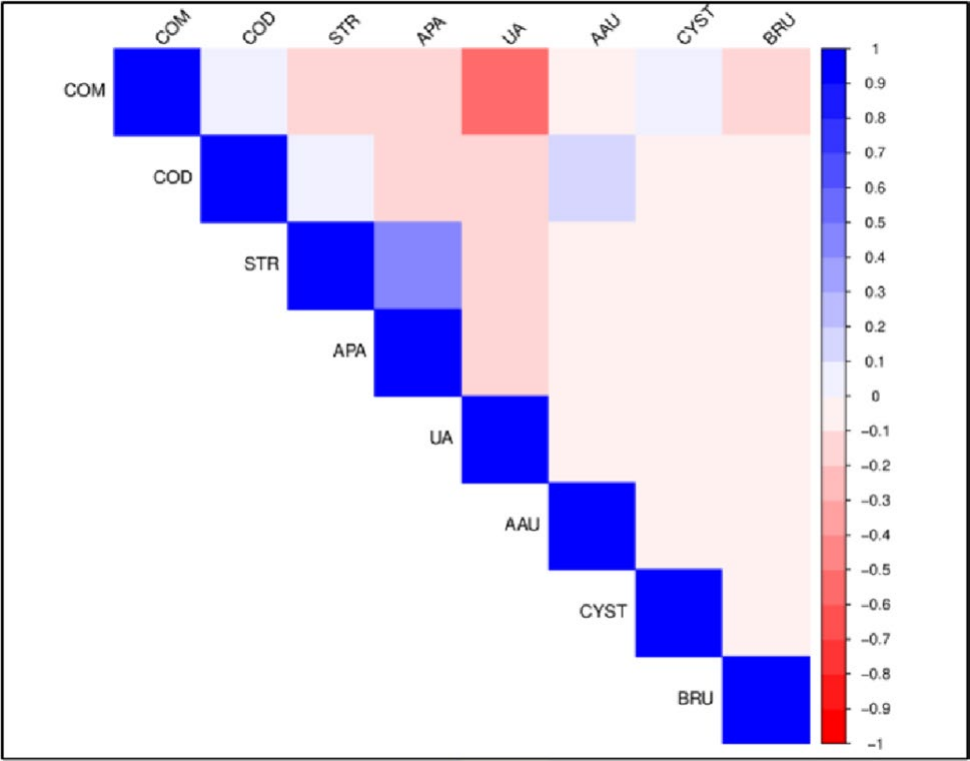
Correlation plot of stone composition in black African males

**Fig. 2:**
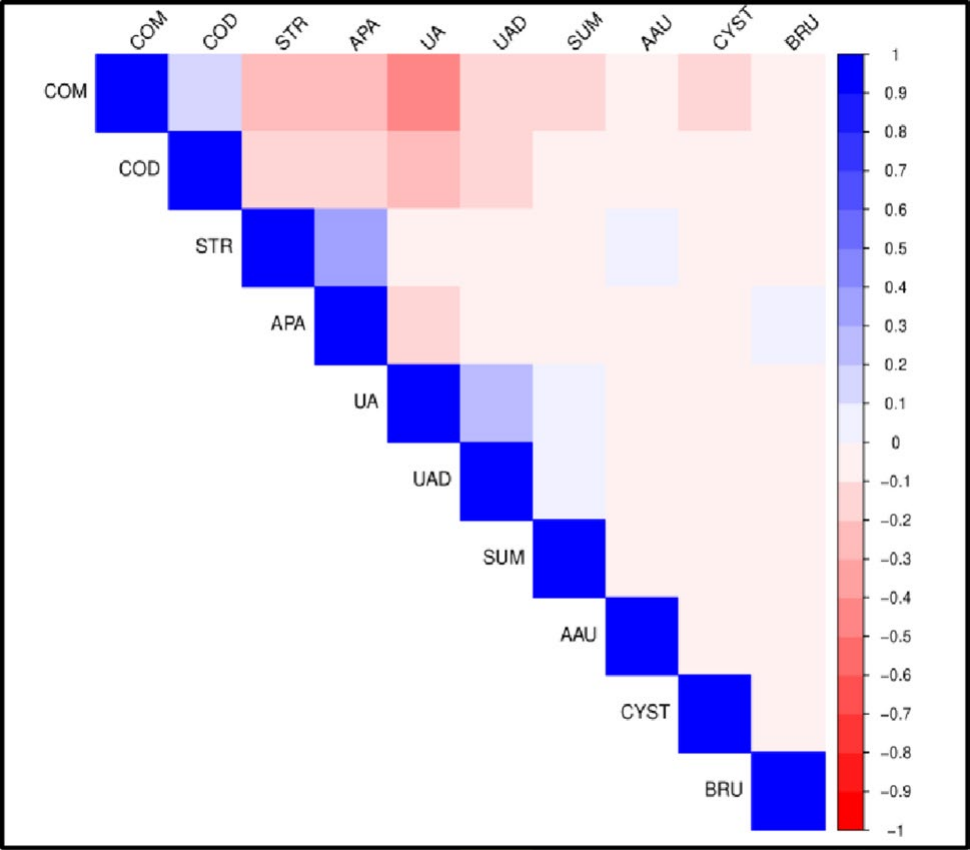
Correlation plot of stone composition in white males

**Fig. 3:**
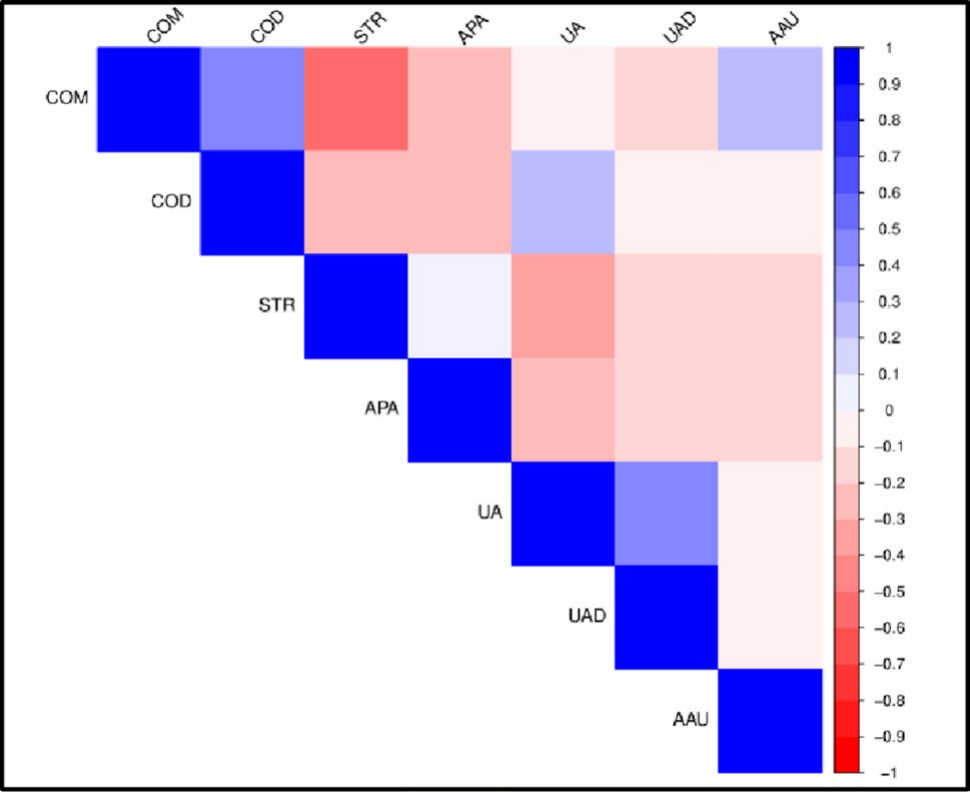
Correlation plot of stone composition in black African females

**Fig. 4:**
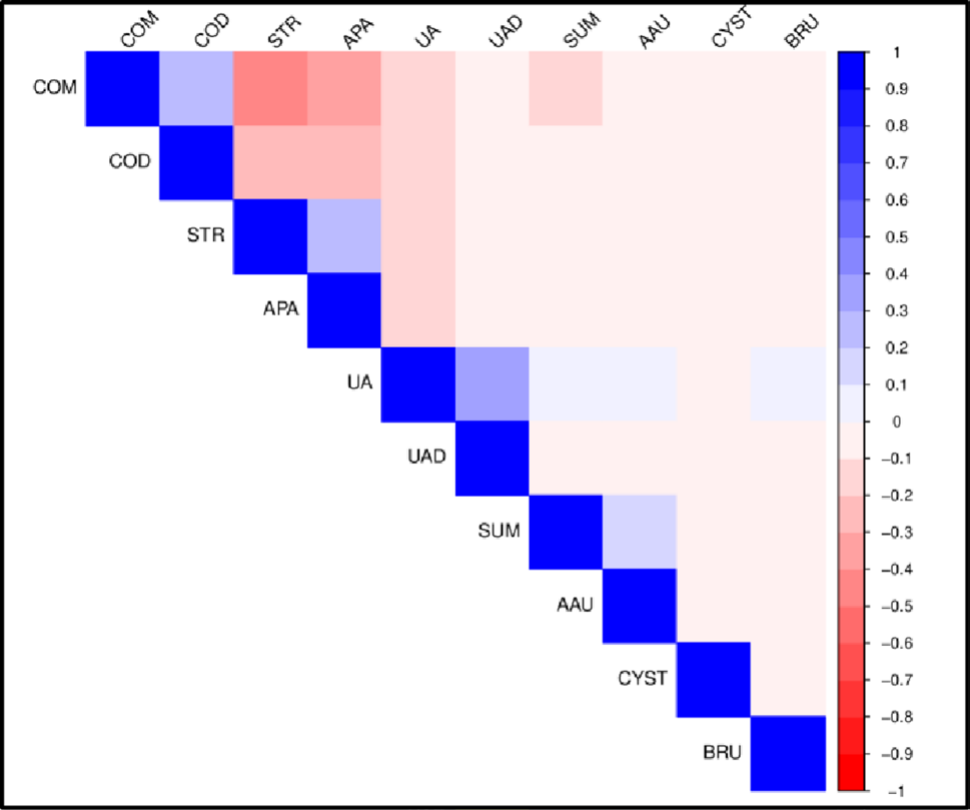
Correlation plot of stone composition in white females

Correlation plots of stone composition in BM and WM are shown in Figs. 1 and 2 respectively while those in black BF and WF are shown in Figs. 3 and 4 respectively. Correlation coefficients and associated p-values are provided in Supplement 1.

In BM (Fig. 1), STR and APA are positively correlated while COM and UA are negatively correlated. In WM (Fig. 2), UA is positively correlated with UAD. COM is negatively correlated with STR, APA and UA while COD is negatively correlated with UA.

In BF (Fig. 3), there are no positive correlations. However, COM and STR are negatively correlated. In WF (Fig. 4), positive correlations occur between COM and COD, between STR and APA and between UA and UAD. Negative correlations occur between COM and STR, between COM and APA, between COD and STR, and between COD and APA.

## Discussion

Our hypotheses in this study were that differences in kidney stone composition between B and W might provide insights into why stones in the former group are relatively rare but in the latter they are relatively frequent, and that these differences might simultaneously enable a better understanding of stone formation in general. Our emphasis per se was to investigate what insights might be obtained from stone composition only.

As described earlier, the only statistically significant inter-race difference in stone composition was the lower proportion of COD stones in B irrespective of gender. This finding is intriguing, given that the proportions of COM stones in B and W were identical. Of importance in the present context is whether this difference provides insight into understanding the different stone occurrence in the two race groups. Possible answers to this question are described in the paragraphs below.

Regarding physicochemical mechanisms, a recent study by Guerra et al. [25] showed that a low COD/COM compositional ratio in individual stones is associated with a relatively lower risk of stone recurrence. Intriguingly, they found that patients exhibiting low values in their stones for this ratio, did not reveal any association with urinary metabolic abnormalities. In the present study, we defined our stones on the basis of the presence of a major component only (> 50%), so we do not have COD and COM compositional data for each stone to enable comparison with Guerra’s observations. To overcome this shortcoming, we adopted a different model in which we speculated that comparison of *proportion* ratios could be regarded as analogous to comparison of *compositional* ratios. Using this approach, our COD/COM proportion ratio in B is 8/57 = 0.14 and 692/1951 = 0.35 in W. Since B is the lower-risk group by virtue of its historically lower stone prevalence than W, our finding of a lower proportion ratio in B supports Guerra’s observation. Unfortunately Guerra and co-workers were not able to offer an explanatory mechanism for their COD/COM-stone risk observation. However, we suggest that the kinetics of COD-COM crystal transformation [26] and weaker crystal-cell adhesive forces involving COD [27] can be invoked to explain the COD/COM phenomena observed by Guerra and ourselves, thereby reinforcing the notion that these physicochemical processes are key role-players in kidney stone formation.

Of interest is to explore whether pathophysiological mechanisms might provide insights into accounting for other observations in our study [28]. An important example is the metabolic syndrome (MS) [29, 30]. This is a cluster of metabolic conditions (obesity, hypertension, impaired fasting glucose, high triglyceride, low HDL cholesterol, type 2 diabetes, T2DM), originally developed for identifying the risk of cardiovascular disease but now widely applied to explain the pathogenesis of several non-communicable conditions [31, 32]. Researchers have noted that some of these traits (overweight, obesity, T2DM and others) are common in calcium oxalate and uric acid stone formers [29, 30, 33].

In South Africa, prevalence of obesity is lower in black African males (BM) than in white males (WM) [34, 35]. Among females, the prevalence of obesity in BF is greater than in WF [34, 35]. With regard to MS, inter-gender studies within each race group (BM vs. WM and BF vs. WF) appear to be rare, as is evident from a recent review [36] in which only one such study was cited [37]. This showed the prevalence of MS to be lower in BM than WM but it was equal in BF and WF. Given that there is an established association between MS and stones, the lower prevalence of MS in B than in W (albeit in males only) indicates a potential contributory factor towards explaining the lower historical prevalence of stones in B.

A possible link associating MS and stones in the South African context, is our observation that uric acid dihydrate (UAD), sodium urate (NaUr) and ammonium acid urate (AAU) stones are absent in B, but are each present in W. This is important for three reasons.

Firstly, UAD is particularly common in patients with type 2 diabetes (T2DM) and obesity [38], both of which contribute towards the MS as mentioned earlier. Secondly, in a recent review of the prevalence of diagnosed T2DM in South Africa’s population groups, the lowest prevalence of this disease occurred in black Africans [39]. These two factors encourage us to suggest that the scarcity of UAD stones in B and their lower levels of obesity, T2DM and MS may be collectively linked to the B vs. W stone anomaly in South Africa. Thirdly, NaUr and AAU have been shown to be present in Randalls plaque (RP) in 3.4% and 0.06% of CaOx stones respectively [40, 41]. RP is a subepithelial calcium phosphate (apatite) deposit at the kidney papilla, formed by a process of heterogeneous nucleation which is widely regarded as the substrate on which calcium oxalate stones are formed [42]. It has been suggested that the presence of NaUr and AAU (as well as other substances) in RP most likely reflects different formation mechanisms [40].This is supported by evidence that the frequent association of NaUr and apatite in RP can be attributed to an intergrowth between these substances and that NaUr is able to serve as a heterogeneous nucleator for apatite [43]. It has also been asserted that it plays a proinflammatory role [40]. These findings strongly suggest that NaUr is a possible initiator of COM stone formation. As such the absence of NaUr and AAU stones in our B collection and their presence in our W collection provides a convincing case for this being a contributory factor in explaining the South African stone anomaly on the one hand and for confirming the notion of their promotive role in stone formation globally, on the other.

Remarkably, one of the earliest studies of RP was conducted by Vermooten in autopsy on B and W South African patients at the Johannesburg General Hospital in 1937 [44]. He found that RP was present in only 4.3% of 699 B, but in 17.2% of 280 W. As far as we are aware, RP research in South African B and W subjects has not been pursued. Nevertheless, the significantly lower prevalence of RP in B compared to W (albeit that the study which reported this finding was conducted more than eight decades ago) suggests another feasible explanation for the South African B vs. W stone anomaly.

Our other findings warrant brief comment. Regarding inter-gender comparisons of stone proportions within the same race group, we observed that three differences were common to both ethnic groups (Results, 4th paragraph). These are consistent with global observations [9–13]. As such, these differences are not relevant in the context of the present study. Additionally, three other inter-gender differences occurred, but only in W (Results, 5th paragraph). These too are consistent with global observations [9–11]. Although the paucity of stones in WF precluded us from investigating why these differences occurred in WM only, we nevertheless speculate that these differences are not relevant in the context of the present study.

Similarly, with respect to same-gender correlations across the race groups, (BM vs. WM and BF vs. WF), a meaningful comparison between BF and WF was not possible because of the small number of WF stones in our study. However, we noted a statistically significant difference involving the correlation (positive) between STR-APA in BM but the absence of the same correlation in WM. This finding indicates that STR and APA are more often both present or both absent in stones originating in B than in W. This is not surprizing since APA is the most frequent co-precipitate with STR in two-component stones [9]. Since STR stone formation is exclusively associated with bacteria that produce the enzyme urease [45] we believe that this inter-race difference is likely to be infection-related. Our literature searches show that all other correlations in the present study are consistent with the composition of stones globally. As such, we regard them as not relevant in the context of the present study.

We acknowledge limitations in our study. Firstly, stones were sourced from two hospitals. As such, the ratios of stone-types associated with the two ethnic groups might have been biased. This needs to be recognized as a potential confounding factor. Secondly our laboratory records did not include data on stone frequencies. This precluded us from investigating the anomaly per se during that period. Such data would have added value to the study. Thirdly, our study is based on our kidney stone collection sourced from two local hospitals. As such it cannot be considered representative of the South African population as a whole. Fourthly we recognise that renal crystallization per se is strongly influenced by multiple factors which include habitual dietary practices. This should be kept in mind when considering our conclusions which are described below.

## Conclusion

Our results have identified promising areas for future investigation which might potentially lead to a better understanding of the intrigues of B vs. W stone-formation locally and for stone-formation more generally. Based on these results, we hypothesize that the smaller proportion of COD in B might be indicative of mechanisms involving the transformation kinetics of COD to COM and the presence of weaker crystal-cell adhesion forces associated with COD. Independently of this pathway, we also propose that the absence of UAD, NaUr and AAU in B is indicative of a loss of promotive capacity for the formation of RP and a concomitantly lower stone occurrence rate in B. We also suggest that the South African stone anomaly might be related to lower MS in B. This is in alignment with the widely held view that MS is a significant and independent risk factor for the development of kidney stones. We conclude that these mechanisms warrant future study for gaining a better understanding of the perplexities of kidney stone formation in local and global settings.

## Data Availability

All the data analysed during the study are available from the corresponding author.

## General

B: Black African subjects (South Africa)
BM: Black male (South Africa)
BF: Black female (South Africa)
MS: Metabolic syndrome
RP: Randall’s plaque
RSS: Relative supersaturation
T2DM: Type 2 diabetes
W: White or White subjects (South Africa)
WF: White female (South Africa)
WM: White male (South Africa)

## Stone components

AAU: Ammonium acid urate
APA: Apatite
Bru: Brushite
CaOx: Calcium oxalate
COD: Calcium oxalate dihydrate
COM: Calcium oxalate monohydrate
CYS: Cystine
NaUr: Sodium urate
STR: Struvite
SUM: Sodium urate monohydrate (equivalent to NaUr)
UA: Uric acid
UAD: Uric acid dihydrate
